# “Circumnavigation”: A Worthy Descriptor to Depict Torrential Mitral Regurgitation and a Reverence to Ferdinand Magellan

**DOI:** 10.7759/cureus.32206

**Published:** 2022-12-05

**Authors:** Robert Biederman, Anantha Sriharsha Madgula, Eric Bucklew, Nazli Okumus, Kartikeya Kashyap

**Affiliations:** 1 Cardiovascular Disease, Allegheny Health Network, Pittsburgh, USA; 2 Internal Medicine, University of Connecticut School of Medicine, Farmington, USA; 3 Cardiology, Allegheny Health Network, Pittsburgh, USA

**Keywords:** magellan, systolic anterior motion of mitral valve, circumnavigation, severe mitral regurgitation, hypertrophic cardiomyopathy

## Abstract

“Circumnavigation” is never used as a verb in cardiology. Hypertrophic obstructive cardiomyopathy is a complex entity that often involves the mitral valve leading to eccentric mitral regurgitation. Utilizing multimodality imaging, assessing the severity of mitral regurgitation, and phenotyping the type of myocardial hypertrophy are achievable with certainty. We describe a case of a 42-year-old male who presented with palpitations and was ultimately diagnosed with hypertrophic obstructive cardiomyopathy with a mitral regurgitation. The torrential mitral regurgitation jet was so severe that it was “circumnavigating” the left atrium. In addition, we also draw historical parallelism with Magellan’s heroic “circumnavigation” of the globe as we celebrate 500 years of his journey. Furthermore, we also describe the multimodality assessment of hypertrophic obstructive cardiomyopathy utilizing transthoracic echocardiography and cardiac magnetic resonance imaging. We discuss the challenges in quantifying such severe mitral regurgitation with any individual imaging modality.

## Introduction

Hypertrophic cardiomyopathy (HCM), previously thought to be a rare disease, is now estimated to have a prevalence of one in 200 to one in 500 [1]. It is a complex disease with multiple anatomical and physiological idiosyncrasies, with mitral regurgitation (MR) being one of the many components. We present the case of a 42-year-old male who presented with dyspnea and palpitations and was ultimately diagnosed with HCM. Assessment of MR in HCM can be challenging given its usual eccentricity due to systolic anterior motion (SAM) of the valve itself, or the subvalvular apparatus. Using multiple imaging modalities could be of great help while assessing this MR, including transthoracic echocardiography (TTE) and cardiac magnetic resonance imaging (MRI) [1,2]. Quantifying MR is essential in HCM as this has several therapeutic implications. We also aim to draw historical parallelism to describe severe eccentric torrential MR using a novel, yet not so new, adjective used to describe MR: “circumnavigate.”

## Case presentation

**Figure 1:**
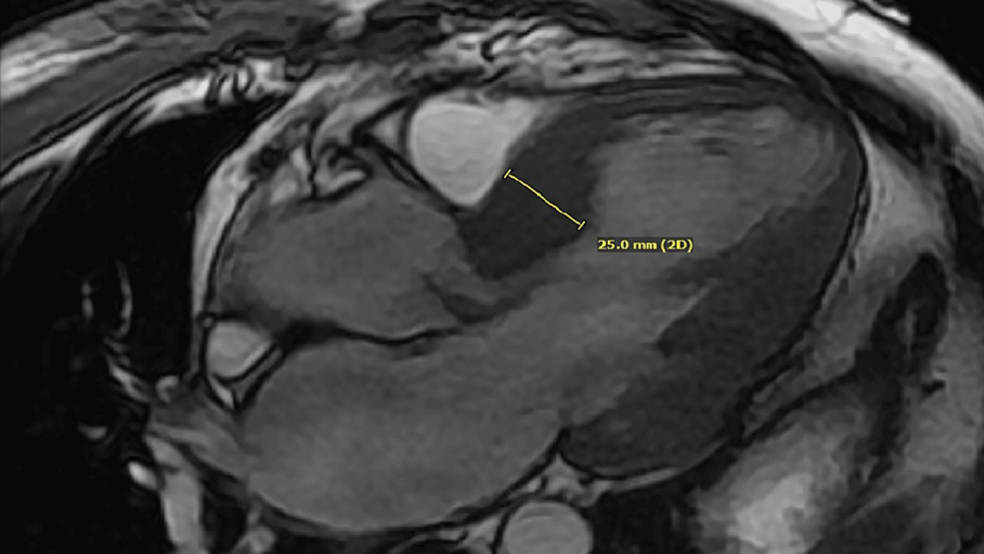
A three-chamber SSFP sequence. Interventricular septum is being measured at its maximum dimension measuring about 25 mm. SSFP, steady-state frequency precession

**Figure 2:**
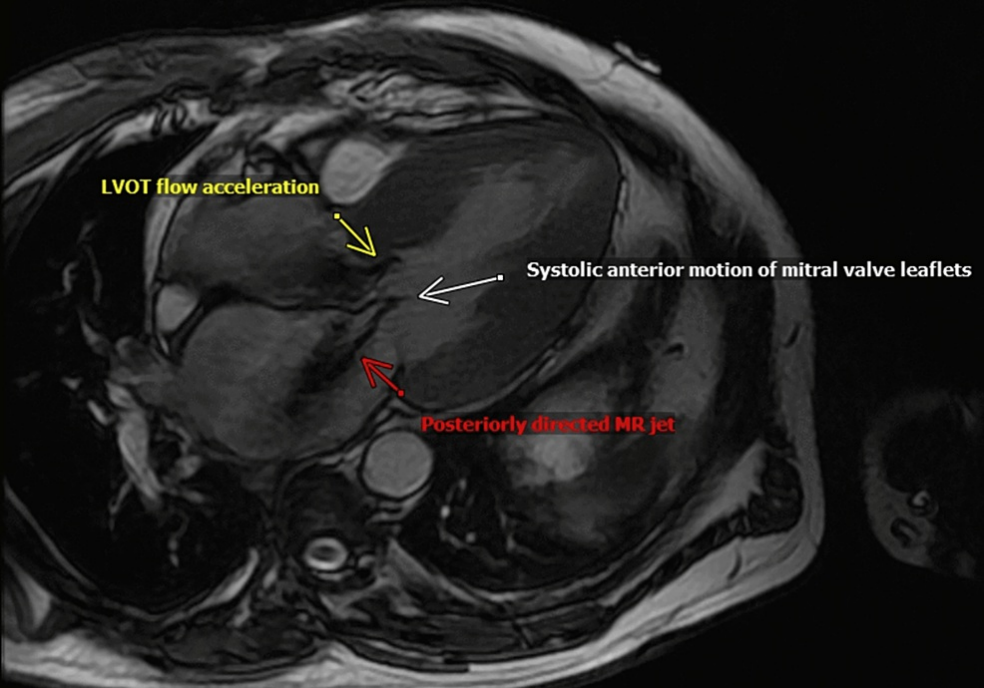
A three-chamber SSFP sequence demonstrating bileaflet SAM, LVOT flow acceleration, and posteriorly directed MR. SSFP, steady-state frequency precession; SAM, systolic anterior motion; LVOT, left ventricular outflow tract; MR, mitral regurgitation

A 42-year-old male presented with sudden onset dyspnea and palpitations. His heart rate was 160 beats per minute on his smart watch, which prompted him to seek immediate attention. He was found to be in atrial fibrillation with rapid ventricular response and endorsed profound dyspnea on minimal exertion over the previous six months. A TTE was obtained for further evaluation. Several important findings were noted, but chief among them was the presence of a markedly hyperdynamic left ventricle (LV) with apical and mid cavity obliteration with resultant bileaflet SAM. The result of this perturbed physiology generated severe torrential eccentric MR (Video 1). With continuous-wave Doppler, the highest gradient across the LV outflow was measured at 173 mm Hg in a sitting position. With Valsalva, the peak gradient increased to 191 mm Hg. He underwent a cardiac MRI to further evaluate this mitral valve syndrome. The cardiac MRI showed thickening of the basal septum upto 25 mm (Figure 1), with the contralateral wall measuring 14 mm. His anterior mitral leaflet measured 33 mm and was elongated. In addition, bilateral SAM was confirmed (Figure 2). Late gadolinium imaging showed midwall patchy enhancement in the basal anteroseptum and basal to mid inferolateral segments. MR regurgitant volume was 105 mL with a regurgitant fraction of 51%, consistent with severe MR. The patient also underwent a stress echocardiogram that showed a rise in LV outflow gradient to 219 mm Hg with exercise giving him an ultimate diagnosis of HCM given the constellation of bilateral SAM, LV hypertrophy, and patchy late gadolinium enhancement (LGE) of his septum. Our patient was referred for septal myectomy given his severe symptoms and elevated gradient.

**Video 1:** Apical four-chamber window from TTE demonstrating severe eccentric torrential MR "circumnavigating" the left atrium. TTE, transthoracic echocardiogram; MR, mitral regurgitation

## Discussion

HCM is an inherited disease of the heart that can result in hypertrophy of the LV myocardium and mitral valve disease that can present with SAM, elongated mitral valve leaflets and MR, aberrant papillary muscle anatomy with bifid or numerous papillary muscles with abnormal chordal anatomy, abnormal septal curvature leading to abnormal flow dynamics, and MR [1]. Evaluation of the heart using multiple imaging modalities including TTE and cardiac MRI can be of great use.

TTE evaluation of HCM begins with the identification of the thickened myocardium, usually the septum. Maximum LV thickness of 15 mm or more at any site is consistent with HCM [2]. Dynamic LV outflow obstruction can be seen with color Doppler demonstrating flow acceleration in the form of aliasing in the parasternal long-axis window in the LV outflow. When this is seen, obtaining gradients using pulse wave Doppler in the apex, mid, and basal regions of the LV, and the LV outflow tract can help with identifying the location of dynamic obstruction [3]. When the velocity across this dynamic obstruction is severe, there may be aliasing with pulse wave Doppler and hence continuous wave Doppler could be used. This is usually done in the apical five-chamber view in order to align our probe as parallel to the flow as possible in order to avoid underestimation. Care must be taken to avoid mixing of the MR waveform with our LV outflow waveform. Change in gradient with Valsalva and positional changes must be documented. When unclear, an exercise stress echocardiogram could also be implemented to provoke a higher gradient [1].

Cardiac MRI can add immense value to the evaluation of HCM. While a TTE can identify pressure gradients and color jets, the spatial resolution of cardiac MRI is unparalleled. Exact thickness of the LV can be measured using the steady-state free precession sequence. Furthermore, we can assess SAM and flow acceleration across the LV outflow using the same sequence [4]. Using three-dimensional volume assessment and phase velocity mapping techniques, we can also assess the mitral regurgitant fraction with precision [5]. Using gadolinium, we can also assess myocardial fibrosis, which adds prognostic value [6]. All this information can help guide the management of the patient and can guide the surgeon if septal myectomy is planned. Perhaps, cardiac MRI’s unique attribute is its’ inherent ability to quantitate MR through non-geometric approaches, not reliant on proximal isovolumetric surface area, and to be more accurate than transthoracic or transesophageal echocardiogram, often leading to changes in patient care [5,7].

Our patient's MR, when assessed using color Doppler on TTE, was seen to “circumnavigate” the left atrium. The MR was directed posteriorly in such an extreme angle and velocity that the regurgitant jet occupied not only greater than 60% of the total left atrial area but also seemed to bank off the dome of the left atrium (LA), angling medially and redirected toward the mitral valve, effectively “circumnavigating” the LA. However, “circumnavigation” is a unique term that has never before been utilized to describe MR. Indeed, “circumnavigation” had been heretofore universally used to solely to describe one penultimate observation: the Herculean effort of Ferdinand Magellan to circumnavigate the globe in 1522.

History of “circumnavigation”

Magellan, an adventurous Portuguese sailor who studied navigation and astronomy, having lost favor with the Portuguese king, King Manuel I, defected to Spain with hopes of completing that work which Christopher Columbus had failed to complete in 1492, namely, to find the Spice Islands. Though that was not his ultimate goal, he was convinced that by sailing west instead of going east as the Portuguese, by the Treaty of 1494, owned all lands to the east. Magellan, generations before his time, believed that he could map out a new route to Indonesia and India and, by finding the Spice Islands, claim certain fame and even fortune [8,9]. Magellan set sail from Spain in 1519 in five bedraggled ships that the Spanish Crown easily parted with having little faith that he would not double cross and return to Portugal. He embarked with supplies for 265 men and began looking for a passage to the West. Multiple ill-fated attempts to probe for a passage for months failed to find the route to the West that would never be found. Magellan, only by passing to the southern-most tip of South America, Tierra del Feugo (Land of Fire), a precarious and deadly route though what became the Straits of Magellan, did he find himself looking at the suddenly placid body of water that he then named “Pacific” Ocean. From there, he made his way over the open ocean that had never before been traversed. As in Coleridge, he “was the first to burst upon that silent ocean.” And, indeed, like the Ancient Mariner’s Doldrums, the journey was fraught with months without food, fresh water, and wind. Finally, after averting with draconian measures, a mutiny by the four other captains for which they were beheaded and quartered, he regained control of the ships only to lose one to Portuguese marauders and another eventually getting stranded. He made his way eventually to the Philippines where he eventually lost his life intervening in a then ill-fated attempt to convert the Indians to Christianity - only to know centuries later that there is a large faction of Christians that can trace their descendants to his efforts - for which he died having succumbed to a poison arrow to the foot [8,9].

His colleagues endeavored to retreat from the Philippines, losing another ship and then finally another before they limped their way back to Spain but not before collecting samples of precious spices, discovering a new galaxy named the Magellanic Clouds in the Southern Hemisphere, and mapping a new route for European trade and the first to set the stage for a modern global economy. Limping into Spain with just 19 sailors and just one leaking dilapidated ship, Victoria, Magellan’s fledgling crew had achieved what Columbus could not [8,9]. The story does not end there. Having circumnavigated the globe, they arrived one day late, or so they thought. Upon their return, the acting captain was surprised to find his calendar off by a day. Through storms, battles, mutinies, horrendous loss of life, and innumerable adversities, the ship's log had been meticulously maintained, as had a second expedition's calendar. Despite this, there was no denying the discrepancy in dates; the expedition's logs showed they had returned to Spain on September 6, 1522, when in fact they returned on September 7, 1522. Their single remaining ship unknowingly crossed what is now known as the International Date Line on their voyage. This critical line is more than an imaginary pen line on a map. In so circumnavigating the globe, the intrepid voyagers crossed from one time zone to another time zone, always moving back by an hour. Upon their return to Spain, having traveled 60,000 miles to circumnavigate the approximately 25,000 mile journey around the globe, they had unknowingly traveled through all the 24 yet-to-be-described time zones and, unbeknownst to them, had rolled back their clocks by a whole day. As they did not know to advance the clock by an hour each time, or in aggregate, their calendar by a day when they crossed the date line, they effectively “lost” 24 hours, one for each of the time zones they crossed in their circumnavigation. Even the Pope could not explain it, but in short order it was clear that Magellan had encircled the globe and proven conclusively that the earth was not flat [8,9].

## Conclusions

Though, not as exciting as Magellan’s circumnavigation of the globe, torrential MR circumnavigating the left atrium epitomizes the term and is a worthy descriptor to depict unequivocal severity of MR. While “coanda effect” is sometimes used to describe this finding, the term fails to capture the enormity of such eccentric torrential MR depicted far more vividly with “circumnavigation.” Fitting in this description is the irony that Magellan circumnavigation occurred almost 500 years ago; we do not have to wait so long to invoke his namesake’s accomplishment to denote torrential MR.
